# All-Fiber Laser Curvature Sensor Using an In-Fiber Modal Interferometer Based on a Double Clad Fiber and a Multimode Fiber Structure

**DOI:** 10.3390/s17122744

**Published:** 2017-11-28

**Authors:** Ricardo I. Álvarez-Tamayo, Manuel Durán-Sánchez, Patricia Prieto-Cortés, Guillermo Salceda-Delgado, Arturo A. Castillo-Guzmán, Romeo Selvas-Aguilar, Baldemar Ibarra-Escamilla, Evgeny A. Kuzin

**Affiliations:** 1 CONACYT—FCFM, Universidad Autónoma de Nuevo León, Av. Universidad S/N, Ciudad Universitaria, San Nicolás de los Garza, Nuevo León 66455, Mexico; rialvarez@conacyt.mx; 2 CONACYT—Optics department, Instituto Nacional de Astrofísica, Óptica y Electrónica, L. E. Erro 1, Sta. Ma. Tonantzintla, Puebla 72824, Mexico; 3 FCFM, Universidad Autónoma de Nuevo León, Av. Universidad S/N, Ciudad Universitaria, San Nicolás de los Garza, Nuevo León 66455, Mexico; pattyprieto@hotmail.com (P.P.-C.); guillermo.salcedadl@uanl.edu.mx (G.S.-D.); arturo.castillogz@uanl.edu.mx (A.A.C.-G.); romeo.selvasag@uanl.edu.mx (R.S.-A.); 4 Optics Department, Instituto Nacional de Astrofísica, Óptica y Electrónica, L. E. Erro 1, Sta. Ma. Tonantzintla, Puebla 72824, Mexico; baldemar@inaoep.mx (B.I.-E.); ekuz@inaoep.mx (E.A.K.)

**Keywords:** fiber optic laser sensor, modal in-fiber interferometer, curvature measurement, erbium-doped fiber laser

## Abstract

An all-fiber curvature laser sensor by using a novel modal interference in-fiber structure is proposed and experimentally demonstrated. The in-fiber device, fabricated by fusion splicing of multimode fiber and double-clad fiber segments, is used as wavelength filter as well as the sensing element. By including a multimode fiber in an ordinary modal interference structure based on a double-clad fiber, the fringe visibility of the filter transmission spectrum is significantly increased. By using the modal interferometer as a curvature sensitive wavelength filter within a ring cavity erbium-doped fiber laser, the spectral quality factor Q is considerably increased. The results demonstrate the reliability of the proposed curvature laser sensor with advantages of robustness, ease of fabrication, low cost, repeatability on the fabrication process and simple operation.

## 1. Introduction

In-fiber modal interferometers used as optical fiber spectral filters have been of persistent interest for their application in optical communication systems, optical instrumentation, and fiber lasers design. Specially, they have been attracted wide attention in fiber sensing because their many advantages such as high sensitivity, immunity to electromagnetic field, small size, low-cost, low maintenance required, and long term operation. Different approaches of fiber sensors designed with in-fiber structures used as wavelength filter as well as sensing element has been reported to measure refractive index (RI) [1,2,3], curvature [4,5,6,7], temperature [8], displacement/strain [9,10], and simultaneous or different physical parameters with the same configuration [11,12,13,14]. In particular, in-fiber curvature sensors have been of increasing interest for applications such as monitoring of smart and composite engineering structures, robotics, prosthetics design, medical treatment, and industrial metrology, among others.

An in-fiber structure based on modal interference used as sensing device requires a fiber element to recombine the excited coupled modes from cladding with the core modes producing an interference effect leading to a modulated transmission of the input signal. The produced interference effect can be caused by tapered fibers [5,11,15], core-offset splices and core diameter mismatch [4,8,13,16], multimode interference (MMI) [2,6,7,10], and multipath by micro-structured fibers [9,14,17], among others. All of the proposed techniques exhibit their own disadvantages. In-fiber structures constructed by tapered fibers require specialized high cost equipment for their fabrication and present fragility of the tapered region which reduces the long-term operation. The core-offset structures have shortage in repeatability of construction method as well as the in-fiber structures formed with micro-structured fiber (because of collapsed splicing zones), with additional disadvantage of high cost of fabrication or affording from accessible commercial fibers. In this regard, in-fiber modal interferometers based on double cladding fibers (DCF) have been demonstrated their reliability with advantages such as robustness, repeatability, long-term operation and ease of construction. To our knowledge, only a few approaches on fiber sensors by using DCF structures have been reported. F. Pang in ref. [3] demonstrated an in-fiber Mach-Zehnder interferometer (MZI) consisting of a single mode fiber (SMF) segment spliced between two DCFs and its application as a RI sensor by transmission spectrum at the 1.3 µm region. Slodeev in ref. [18] studied the transmission spectrum under bending of an in-fiber DCF structure. In both reported investigations, the proposed structures exhibit transmission spectrum response with wavelength shifting and fringe visibility changes depending on the measured parameter, which make them useful for fiber sensing. Their main disadvantage is the low fringe visibility of the transmission spectral response at the 1.5 µm wavelength range. Nevertheless it can be useful for multi-wavelength lasers design. Recently, we reported a switchable dual-wavelength erbium-doped fiber laser (EDFL) using a short wavelength period in-fiber MZI based on a DCF as spectral filter [19]. However, the design of fiber laser sensors by wavelength displacement requires spectral filters with long free spectral ranges (FSR) and high fringe visibility.

In this paper, we propose the design of an in-fiber modal interferometer with transmission spectrum characteristics of high fringe contrast and long FSR, suitable in its application as spectral filter for fiber laser sensors operating by wavelength displacement of the generated laser line with improvement of the Q value and tunable fiber lasers design. The construction of the in-fiber structure is based on a conventional SMF-DCF-SMF (SDS) structure. In addition, a multimode fiber (MMF) is inserted between the input SMF and the DCF in order to increase the fringe visibility of the transmission spectrum. An in-fiber spectral filter with long wavelength period of ~21.39 nm and insertion losses of ~29% is achieved. In addition, we propose and experimentally demonstrate a curvature laser sensor by using the in-fiber structure as a spectral filter and as well as a sensing element inserted in a ring cavity EDFL. By wavelength displacement of the generated laser line, sensing of the curvature is obtained in a range of ~11 nm with narrow bandwidth of ~0.251 nm and high optical noise-to-signal ratio (ONSR) of ~43.85 dB. The sensor sensitivity to curvature is of −8.156 nm/m^−1^ in a curvature range of 1.523 m^−1^. The proposed all-fiber laser sensor exhibit advantages of high Q value, easy interrogation, and high intensity.

## 2. Operation Principle and Characterization of the In-Fiber Structure

**Figure 1:**
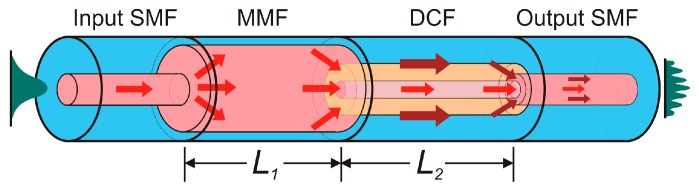
Schematic diagram of the in-fiber SMDS structure.

The three-dimensional schematic of the proposed in-fiber structure is shown in Figure 1. The in-fiber device was constructed by fusion splicing of a SMF-MMF-DCF-SMF (SMDS) structure. The design of the optical device is based on a DCF to cause modal interference [18,19]. The DCF segment, with length of $L_{2}=2.5$ cm, is a homemade W-profile fiber with diameter of core, inner cladding and outer cladding of 7, 47 and 125 µm, and average RI of 1.459, 1.454 and 1.457, respectively. In the first splice of a conventional SDS structure, power from the core mode of the SMF is distributed between modes of the DCF because of the different RI profiles when light is injected from the input SMF to the DCF with different parameters of the cores. Due to the depressed-inner-cladding profile of the DCF, the cladding modes are not restricted to the inner cladding. Cladding modes from inner and outer cladding are returned to the core-cladding region and propagated along the DCF from the first to the second splice. Some part of the light is scattered by transfer energy to high-order modes and losses at the outer cladding-surface interface [18]. At the second splice, the core mode and excited cladding modes LP_0*m*_ propagated through the DCF section interfere at the SMF core where cladding modes of the DCF are converted into the core mode LP_01_ of the output SMF. Additionally at the second splice, part of the light is coupled to the cladding of the SMF without returning to the core.

Then, the core and the cladding of the DCF act as the arms of a MZI interferometer. Since both arms have the same lengths, the phase difference is produced by the effective RI difference of the modes propagated through the DCF. The interference process produces a periodical transmission spectrum as a function of the accumulated phase difference during the length of the DCF and the intensity of the propagated modes. Due to the numerous DCF cladding modes excited and the high-order modes interference, the transmission spectra exhibit some irregular oscillations. However, the average wavelength period of the interference transmission spectrum can be approximated to the case of two-mode interference of a MZI given by [20]:

(1) $$\Delta \lambda =\frac{\lambda^{2}}{L_{2}\left[n_{co}-n_{cl}+\lambda \frac{d(n_{co}-n_{cl})}{d\lambda }\right]}\;,$$

where $n_{co}$ and $n_{cl}$ are the effective refractive indices of the core mode and the cladding mode of the DCF, respectively. With $n_{eff}=n_{co}-n_{cl}$. Operating in a narrowed wavelength region, the free spectral range can be calculated by [21]:

(2) $$\Delta \lambda ≅\frac{\lambda^{2}}{\Delta n_{eff}L_{2}}\;.$$

To complete the SMDS in-fiber structure, a segment of MMF (Thorlabs AFS105/125Y, core diameter $r_{c}=105\;µm$, cladding diameter of 125 µm, and NA of 0.22) with length $L_{1}=1.505\;\mathrm{cm}$ was included between the input SMF and the DCF. The MMF segment was included in order to improve the fringe visibility of the transmission spectrum by increasing the intensity of the dominant LP_0*m*_ cladding modes, coupling more light to the first cladding of the DCF. However, the insertion of the MMF segment also involves a change of the output transmission spectrum. When light is coupled from the input SMF into the MMF section, the core mode from the SMF is distributed to different excited modes on the MMF. Due to the phase difference between the propagated modes along the MMF length, multimode interference occurs. The output spectrum from the MMF exhibits resonant dips where the transmitted intensity is minimal. According with Mohammed at ref. [22], the phase difference condition to reach constructive interference between two propagated modes LP_0*m*_ and LP_0*n*_ is $\left(\beta_{m}-\beta_{n}\right)/L_{1}=2\pi N$, where $\beta_{m}-\beta_{n}$ is the difference in the longitudinal propagation constants between the two modes. The wavelength at which a resonant dip with maximum interference occurs is given by [23]:

(3) $$\lambda_{v}=\frac{16n_{c}r_{c}^{2}N}{\left(m-n\right)\left[2\left(m+n\right)-1\right]L_{1}}\;\left(m>n\right),$$

where *m* and *n* are the roots of the zero-order Bessel function and N is an integer. The wavelength spacing between two adjacent dips is obtained from Equation (3) with N=1.

The SMF-MMF section of the SMDS structure modulates the input signal by MMI effect and the transmitted spectrum of this section acts as the input signal at the lead-in facet of the DCF. Because of the high ratio of ~2.23 between the diameters of the MMF core and the DCF first cladding, the intensity of the excited LP_0*m*_ is increased leading to an enhancement of the fringe visibility due to the modal interference.

**Figure 2:**
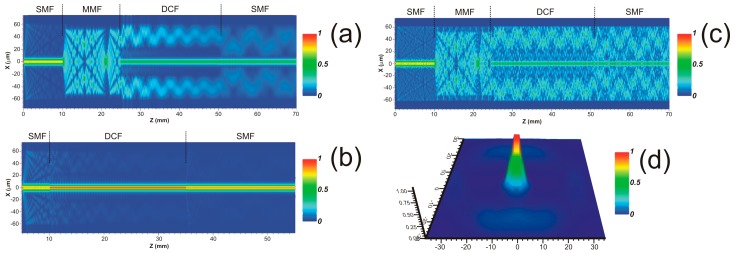
BPM Simulations of intensity distribution in the xz plane for: (**a**) used SMDS; (**b**) SDS structure; (**c**) SMDS without black paint application on the structure surface; (**d**) BMP simulation of the three-dimensional optical field amplitude profile of the SMDS structure at the output SMF.

The light propagation along the in-fiber structure was simulated by beam propagation method (BPM). The light source at the input SMF segment is a normalized core mode input with maximal intensity at the wavelength of 1550 nm. Figure 2a shows the longitudinal amplitude distribution of the input light field in the xz plane. As it can be observed, the energy from the input SMF core mode is distributed in different modes along the MMF segment producing MMI at the lead-in facet of the DCF. Dominant cladding modes propagated along the outer cladding are strongly excited and returned to the cladding-core region by the significant part of the light from the MMF coupled into core and inner cladding of the DCF. The portion of the light coupled from the MMF to the outer cladding is dissipated through partial energy transfer to high-order modes on the outer cladding-surface interface. The most significant intensity is observed along the core. As a result, modal interference is produced by the optical path difference between the propagated core and cladding modes coupled into the core of the output SMF. In addition, some part of the light from the DCF is coupled into the cladding of the output SMF which is highly attenuated along the SMF without returning to the core. In order to compare the SMDS structure with a conventional SDS structure, the mode propagation along the SDS structure (without the MMF) was simulated by BPM in Figure 2b. In case of a SDS structure, the portion of light from the SMF which is distributed into cladding modes of the DCF is highly decreased as a result of the similar core dimensions between the SMF and the DCF. As a result, the interference produced at the output SMF core due to the DCF core mode and the weak cladding modes leads to a modulation of the input signal with lower fringe contrast compared with a SMDS structure. Because of the lengths of the fiber segments forming the SMDS structure which were fusion spliced, the in-fiber interferometer is formed by stripped fiber sections. Therefore, part of the light is scattered in different high-order modes because of the outer cladding-surface RI difference producing oscillations because of their interference. Then, to increase losses for high-order modes and eliminate their interference, the SMSD structure was painted in black [20]. Figure 2c shows the BPM simulation of the SMDS structure without performing the surface painting. In this case compared with simulation results obtained in Figure 2a (were the surface painting was simulated), numerous cladding modes are returned to the cladding-core region due to the lower RI on the surface than the cladding of the different fiber segments. The output field amplitude profile at an output SMF length of 5 cm is shown in Figure 2d. The most significant portion of the light power from the core mode and the excited cladding modes from the DCF is distributed in the SMF core rather than in the cladding.

**Figure 3:**
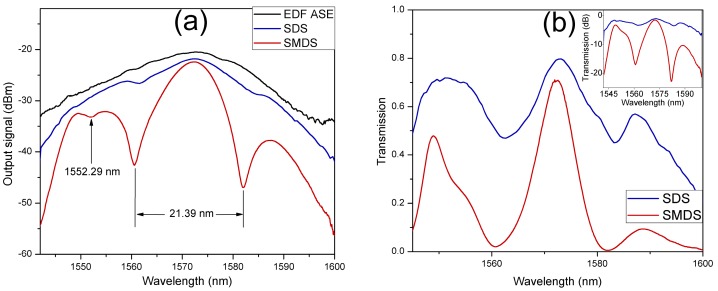
(**a**) Measured output signal of the ASE source, SDS and SMDS in-fiber structures; (**b**) transmission spectral response of the SDS and the SMDS in-fiber interferometers.

The characterization of SMDS interferometer transmission spectrum was performed by using the amplified spontaneous emission (ASE) from an erbium-doped fiber (EDF) as input signal at the input SMF. The output spectrum was obtained at the output SMF by an optical spectrum analyzer (OSA) with resolution of 0.03 nm. Figure 3a shows the measured ASE output signal of the EDF (black curve). In order to compare the operation performance between the SMDS (red curve) and the conventional SDS (blue curve) structures with similar construction parameters, their transmitted output signal due to the ASE input signal were also measured and shown in Figure 3a. As it can be observed, both in-fiber interferometers exhibit periodical transmission spectra with a wavelength period around 21 nm with a maximum transmission peak around 1572.5 nm. For the SMDS structure, a resonant dip is observed at the wavelength of 1552.29 nm as a result of the multimode interference from the SMF-MMF section of the SMDS structure. By using the Equation (3), the calculated wavelength at which the transmission dip occurs is of 1552.11 nm with m=5, n=3, and N=11. The calculated wavelength spacing between two adjacent resonant dips is of 141.1 nm. Figure 3b shows the transmission of both in-fiber modal interferometers. The transmission was estimated as the measured output signal of each structure divided by the measured ASE source output signal. As it can be observed the transmission of the SDS structure (blue curve) exhibits a lower fringe contrast than the obtained for the SMDS structure (red curve). The transmission losses for the SDS and the SMDS structure are of ~21% and ~29%, respectively. As it can be observed in the inset of Figure 3b, the SMDS structure shows a significantly increased fringe contrast from ~2.2 to ~22.3 dB. Is it also noticed that the transmission dip in the SMDS output spectrum is not present in the case of the SDS structure transmission spectrum.

**Figure 4:**
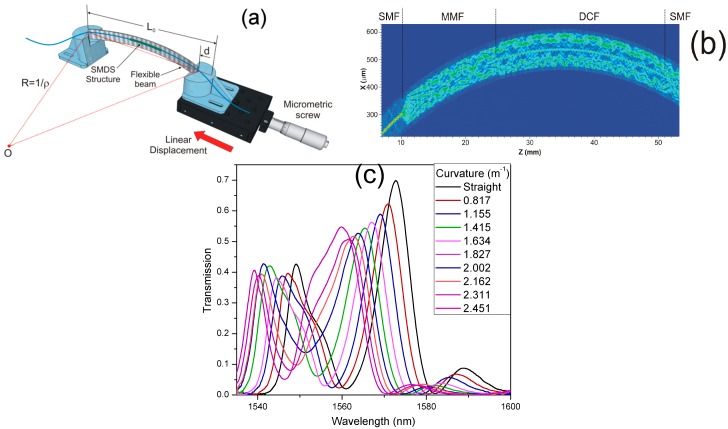
(**a**) Schematic of the mechanical system for curvature application; (**b**) BPM simulation of the bent SMDS structure; (**c**) Transmission of the SMDS structure under curvature variations.

In order to evaluate the bending sensitivity of the SMDS structure, the evolution of the transmission spectrum by curvature application was measured by using the mechanical system shown in Figure 4a. The in-fiber device was mounted on a thin flexible metallic beam whose ends were placed on a pair of homemade plastic stages to ensure its position. One of the stages remains fixed while the other is attached to a micrometric translation stage. Curvature variations were applied to the flexible beam by linear displacements on the translation stage. The fiber curvature (ρ) is approximately calculated by [24]:

(4) $$\rho =\frac{1}{R}≅\sqrt{\frac{24d}{L_{0}^{3}}}$$

The length of the flexible beam $L_{0}=19.3\;\mathrm{cm}$ is the separation between the two translation stages without bending application on the fiber structure and d is the linear movement distance of the translation stage. The SMDS structure is sensitive to curvature variations. Then, when curvature is applied its physical length and the mode RI are varied. The optical path of the cladding modes are modified while the core mode is immune to the curvature variations. The effective RI variation experienced by the interfered modes is approximately the same; therefore, the optical path difference is mainly affected by the physical length leading to a wavelength displacement of the SMDS transmission spectrum as it is shown in the BPM simulation of the SMDS structure under curvature of Figure 4b. When the DCF is bent, a significant part of the cladding modes trying to escape through the inner to outer cladding region will be returned back to the core-cladding region due to the W-profile of the DCF refractive indices. The estimated transmission due to the ASE source of the SMDS structure under curvature variations is shown in Figure 4c. The linear displacement of the translation stage was varied each 200 µm from straight position to 1800 µm, corresponding to a curvature variation of 2.451 m^−1^. When curvature is increased, the transmission spectrum is wavelength displaced toward shorter wavelengths in a range of ~12.5 nm. The resonant dip produces irregular oscillations on the transmission spectrum profile as it is wavelength displaced. However, the maximal transmission wavelength peak remains the same.

## 3. Experimental Setup and Results

**Figure 5:**
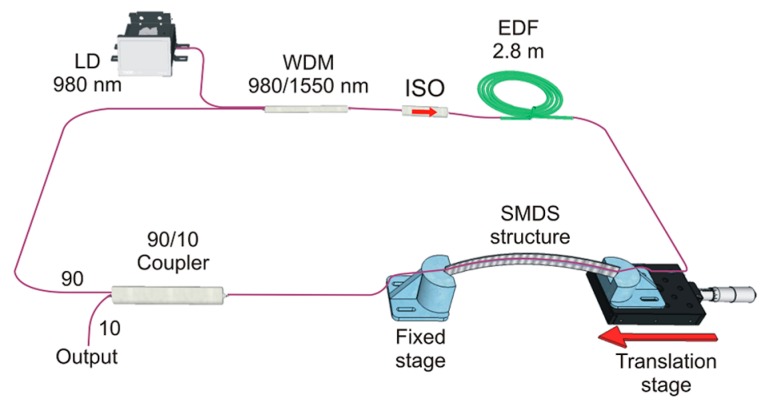
Experimental setup of the fiber ring EDF laser curvature sensor.

The interference spectrum of the curvature sensor exhibits an increased fringe visibility; however, its comprehensive performance is highly improved when the SMDS structure is included within a fiber laser cavity. Figure 5 shows the experimental setup of a proposed ring cavity EDFL including the SMDS structure. The laser cavity uses a 2.8 m long L-band EDF (concentration of 3000 ppm and NA of 0.25) as a gain medium. The EDF is pumped by a 250 mW laser diode at 980 nm through a 980/1550 nm wavelength division multiplexer. An optical isolator (ISO) ensures unidirectional light propagation. The SMDS structure and a 90/10 optical coupler used as the laser output complete the cavity. The laser output spectrum is measured with the OSA at the output port of the 90/10 coupler. The SMDS structure acts as spectral filter and sensing element. The spectrum response of the curvature sensor increases its visibility (by a high ONSR of the laser line) and considerably reduces the full width at half maximum (FWHM).

**Figure 6:**
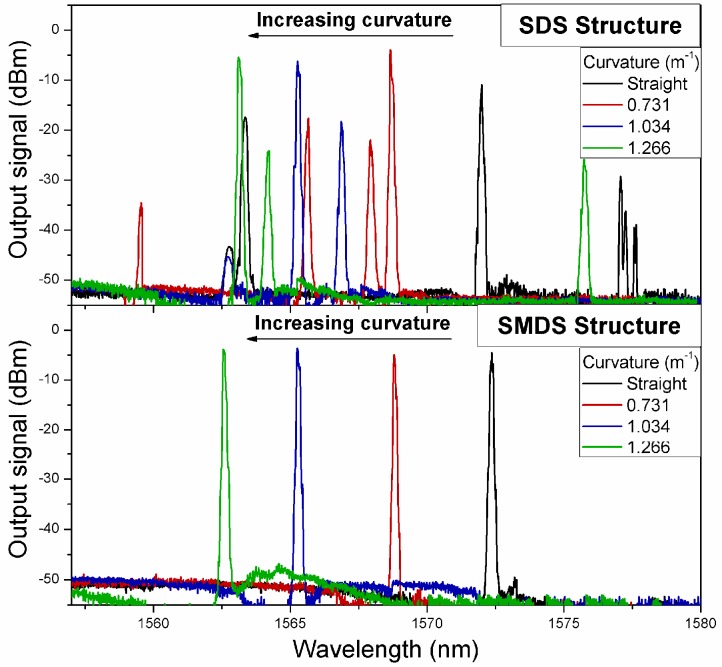
The output signal of the EDFL under curvature variations for SDS and SMDS structures used as spectral filters.

Figure 6 shows the experimental results comparison of the laser performance by curvature application for both SDS and SMDS in-fiber modal interferometers. The output signal of the EDFL was measured at the output port by the OSA for each in-fiber interferometer used as spectral filters. A set of 4 linear displacements on the mechanical system from straight position to 480 µm each 160 µm were performed to apply curvature variations on each structure. As it can be observed, when the SDS structure is inserted within the cavity, different laser lines are simultaneously generated. The EDF is a homogeneous gain medium at room temperature. Then, because of the low fringe contrast exhibited by the SDS interferometer is observed a strong mode competition between different wavelengths on the SDS transmission spectrum with similar gain to generate laser emission. On the other hand, due to a higher fringe contrast exhibited on the SMDS structure, a well-defined maximum peak of its transmission spectrum allows to generate a single laser emission at the wavelength of the maximum peak. It is worth to note that the simultaneously generated laser emission observed with the SDS structure can be reduced (but not eliminated) by using a polarization controller which is not considered within the proposed laser cavity.

**Figure 7:**
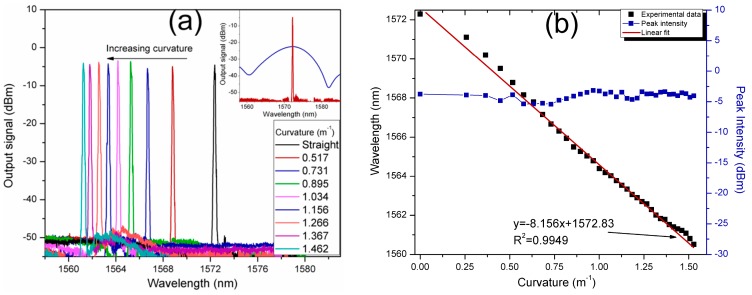
(**a**) The output signal of the laser sensor based on the SMDS in-fiber structure under curvature variations; (**b**) laser peak wavelength and peak intensity as a function of the curvature.

The spectral response of the laser curvature sensor is shown in Figure 7. Curvature changes on the flexible beam were applied by linear displacement of the translation stage from 0 to 700 µm, corresponding to a curvature range from straight position to 1.523 m^−1^. The laser output spectra were measured by the OSA at the output port. As it is shown in the inset of Figure 7a, the SMDS structure acts as transmission spectral filter which selects the preference of the laser emission (red curve) at the wavelength in which the maximal transmission peak of the SMDS structure interference spectrum (blue curve) is reached. Moreover, the generated laser line is wavelength tuned as the interference spectrum is wavelength shifted by curvature variations on the SMDS structure acting as sensing element. As the curvature is increased, the laser line is tuned toward shorter wavelengths in a total tuning range of ~11 nm. The laser wavelength displacement and the peak intensity as a function of the sensor curvature are shown in Figure 7b. The peak intensity varies in a range from −3.17 to −5.42 dBm. The obtained results show a curvature sensitivity of the laser sensor of −8.156 nm/m^−1^ by using a linear fitting with R^2^ of 0.9949. The estimated average FWHM of the laser line is of ~0.251 nm and the OSNR is of 43.85 dB.

**Figure 8:**
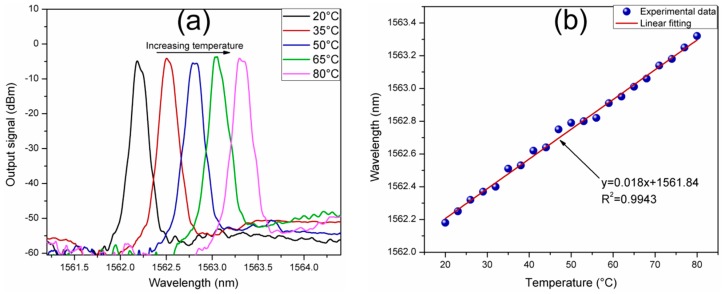
(**a**) The output signal of the laser sensor under temperature variations; (**b**) Laser peak wavelength as a function of the surrounding temperature.

The spectral response of the SMDS structure is sensitive to temperature changes. In order to investigate the laser sensor response as a function of the surrounded temperature, the sensing element was place into an isolated chamber with controlled temperature. Initially, at room temperature, the laser line wavelength was tuned to 1562.3 nm which corresponds to a curvature application of ~1.29 m^−1^. The measured laser spectra with surrounding temperatures of 20, 35, 50, 65 and 80 °C is shown in Figure 8a. With the increase of the temperature, the laser line shifts toward longer wavelengths. The wavelength shift of the generated laser line as a function of the temperature can be linearly fitted with a R^2^ of 0.9943 and a low average temperature sensitivity of 18 pm/°C, as it is shown in Figure 8b. The temperature was varied from 20 to 80 °C with a 3 °C interval. The laser line only displaces 1.14 nm over a temperature range of 60 °C. Although the temperature sensitivity is very low compared to curvature, the effect caused by temperature affects the curvature sensing. In this regard, some methods to guarantee the accuracy such as double parameter matrix method, constant temperature environments or initial calibrations, must be taken into account.

## 4. Discussion

In order to estimate the comprehensive sensing properties of the sensors based on in-fiber modal interference structures, Bai in Ref. [9] defined a quality factor Q value used to describe the quality of the sensor in terms of its sensing sensitivity, resolution and accuracy. The Q value is expressed as:

(5) $$Q=\frac{{\mathrm{KVS}}^{2}}{\mathrm{FWHM}},$$

where S is the sensitivity, V is the visibility and K is a unit coefficient to normalize the physical dimension. As a result, the Q value is a constant without dimension. It is worth to note that a laser based sensor increases the visibility and narrows the FWHM. Thus, laser sensors highly improve the quality factor compared to straight transmission spectrum sensors. Only one in-fiber curvature sensor by wavelength shift based on a DCF has been reported, to our knowledge. However, the results were obtained in the 1.3 µm wavelength region. Then, in order to show the reliability of the proposed sensor, it was compared in terms of the Q value with reported curvature sensors with similar in-fiber structures operating in the 1550 nm wavelength band. The comparison is listed in Table 1. As it can be observed, when the sensor is inserted into a laser cavity, the Q value is highly increased due to the high visibility and the reduced FWHM [7,17]. Compared with the in-fiber sensors designed by core-offset splicing and no-core fiber structures, the proposed sensor exhibits lower sensitivity to curvature which is typical of DCF-based in-fiber sensor [18]. In terms of the Q value, the proposed laser sensor demonstrates an improvement on the sensing quality compared with the most of the reported curvature sensors by using in-fiber structures. In addition, the proposed sensor shows high reliability on an extended curvature range of 1.523 m^−1^. With a design improvement of the laser cavity in terms of the linewidth narrowing, the Q value can be increased as it can be observed from the Table 1 for the results shown in ref. [7].

## 5. Conclusions

In this paper, we experimentally demonstrated a novel curvature laser sensor by using an in-fiber structure formed by MMF and DCF segments. The MMF was added to increase the fringe visibility of the transmission spectrum of the SMDS modal interferometer to obtain an in-fiber modal interferometer based on the use of a DCF, with appropriate characteristics to design a curvature fiber laser sensors based on the wavelength displacement of the laser line. The implementation of a curvature laser sensor significantly increases the Q value of the sensor compared with straight transmission fiber sensors. Compared with the conventional DCF-based in-fiber structure, the fringe visibility of the transmission spectrum was increased from ~2.2 to ~22.3 dB. The SMDS interferometer exhibits a long wavelength period of ~21.39 nm and transmission losses of ~29%. The in-fiber structure was inserted in a ring cavity EDFL acting as spectral filter and sensing element. The curvature laser sensor by wavelength displacement shows sensitivity to curvature of −8.156 nm/m^−1^ in a curvature range of 0.409–1.523 m^−1^, over a wavelength tuning range of ~11 nm.

The generated laser line exhibits a FWHM of ~0.251 nm and ONSR of ~43.85 dB. The use of the in-fiber structure in a fiber laser sensor increases significantly the quality factor Q than an ordinary transmission spectrum curvature sensor. The reliability of the proposed all-fiber laser sensor with advantages such as robustness, high Q value, easy interrogation, and high intensity is experimentally demonstrated.

## Figures and Tables

**Table 1:** Comparison between similar reported in-fiber sensors and this study.

Ref.	Sensitivity (nm/m^−1^)	FWHM (nm)	Visibility (dB)	Q Value	Curvature (m^−1^)	Structure	Notes
[12]	−0.179	0.678	9.84	0.465	3.062–4.050	Hollow core fiber (HCF) SMF + abrupt-taper joints	
[18]	9.6	14	14.63	96.3	0–14	LEAF-DCF-LEAF	@1.3 µm
[6]	−10.38	2	11.53	621.1	0–0.5	SMF-MMF-SMF	
[5]	−13.17	2.45	26.7	1891.7	4.8–6.33	Two cascading abrupt-tapers in SMF	
[4]	−22.95	1.471	24.13	2480.1	0.353–2.812	SMF core-offset splicing	
[14]	−2.55	0.073	46	4097.4	1.231–1.459	Four core fiber (FCF) between SMF	Laser sensor
[7]	−22.33	0.06	42	349040	0.212–0.346	No-core fiber (NCF) between SMF	Laser sensor
This paper	−8.156	0.251	43.85	11621.7	0–1.523	SMF-MMF-DCF-SMF	Laser sensor
